# Roles of a Glycolipid MPIase in Sec-Independent Membrane Protein Insertion

**DOI:** 10.3390/membranes14020048

**Published:** 2024-02-08

**Authors:** Kaoru Nomura, Shoko Mori, Keiko Shimamoto

**Affiliations:** 1 Bioorganic Research Institute, Suntory Foundation for Life Sciences, 8-1-1 Seikadai, Seika-cho, Soraku-gun, Kyoto 619-0284, Japan; mori@sunbor.or.jp (S.M.); shimamot@sunbor.or.jp (K.S.); 2 Department of Chemistry, Graduate School of Science, Osaka University, 1-1 Machikaneyama, Toyonaka, Osaka 560-0043, Japan

**Keywords:** glycolipids, membrane protein, Sec-independent insertion, membrane lipids, physicochemical property of membrane

## Abstract

Membrane protein integrase (MPIase), an endogenous glycolipid in *Escherichia coli* (*E. coli*) membranes, is essential for membrane protein insertion in *E. coli*. We have examined Sec-independent membrane protein insertion mechanisms facilitated by MPIase using physicochemical analytical techniques, namely solid-state nuclear magnetic resonance, fluorescence measurements, and surface plasmon resonance. In this review, we outline the physicochemical characteristics of membranes that may affect membrane insertion of proteins. Subsequently, we introduce our results verifying the effects of membrane lipids on insertion and estimate the impact of MPIase. Although MPIase is a minor component of *E. coli* membranes, it regulates insertion by altering the physicochemical properties of the membrane. In addition, MPIase promotes insertion by interacting with substrate proteins. We propose comprehensive mechanisms for the membrane insertion of proteins involving MPIase, which provide a physicochemical basis for understanding the roles of glycolipids in protein translocation.

## 1. Introduction

**Figure 1:**
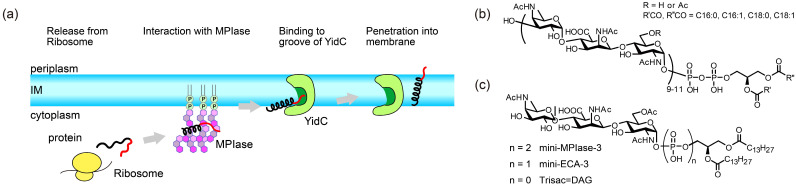
(**a**) Schematic model of the Sec translocon-independent membrane protein integration pathway into the inner membrane (IM) of *E. coli.* (black: hydrophobic region, red: hydrophilic region). MPIase is presumed to function prior to an insertase YidC. (**b**) Molecular structure of MPIase. Approximately one-third of the 6 position on GlcNAc residues is *O*-acetylated. The number of repeating trisaccharide units ranges from 7 to 14, but most are from 9 to 11. (**c**) Molecular structures of synthesized MPIase analogs.

Efficient functioning of membrane proteins relies on their accurate insertion into the membrane. The mechanisms of membrane insertion have been the subject of considerable research. Membrane insertion depends on the size of the protein to be inserted. Large membrane proteins, which are characterized by multiple transmembrane domains, require Sec translocons, which are membrane protein complexes that form pores for proteins to permeate the membrane (Sec-dependent membrane insertion process) [1,2]. Since highly homologous Sec translocons are present in all organisms, the mechanism of Sec-dependent insertion is considered to be basically conserved. In contrast, Sec-independent processes (Figure 1a) have been shown to occur in a subset of small membrane proteins and tail-anchored (TA) membrane proteins that cannot use translocons [3,4]. In eukaryotes, chaperones for membrane targeting of TA proteins have been identified [5,6]. However, because such chaperones have not been found in *Escherichia coli* (*E. coli*), the Sec-independent process in *E. coli* was previously considered to be a spontaneous process. Then, it became evident that spontaneous insertion into liposomes composed only of commercially available *E. coli* phospholipids (EPLs) does not reflect the correct biological phenomena. For example, mannitol permease (MtlA), a Sec-dependent protein, spontaneously inserted into EPL liposomes even without the Sec translocon, whereas such improper insertion did not occur in the inner membrane vesicles [7]. These results suggested that a blocking factor for spontaneous insertion was present in the inner membrane of *E. coli*. Diacylglycerol (DAG) was subsequently identified as a factor that blocks unregulated spontaneous insertion into EPL membranes. DAG is a simple molecule with two fatty acid chains covalently bonded to a glycerol moiety via ester bonds. Nishiyama et al. reported that spontaneous membrane insertion of proteins was significantly inhibited in EPL liposomes containing a physiological concentration of DAG (2–3 *w*/*w*% of EPLs) [7,8]. Furthermore, in a later study, they discovered that a minor component of *E. coli* membranes, membrane protein integrase (MPIase), restored Sec-independent insertions that were suppressed by DAG [7,9,10]. MPIase is also involved in Sec-dependent integration and translocation [7,11]. Despite its name, structural analysis revealed that MPIase is a novel glycolipid composed of a DAG anchor moiety, a long sugar chain consisting of approximately ten trisaccharide units, and a pyrophosphate linker (Figure 1b) [10]. MPIase dose-dependently enhances Sec-independent insertion into DAG-containing liposomes [9].

Moreover, in the Sec-independent insertion process, substrate proteins require the help of a proteinaceous factor called YidC as the hydrophilic proportion of the substrate proteins increases [12,13,14]. YidC transiently captures the hydrophilic regions of a substrate protein in its hydrophilic groove in the membrane (Figure 1a) [15]. In addition, the short transmembrane domain of YidC locally thins the lipid bilayer, facilitating the crossing of the hydrophilic region of the protein through the bilayer [12,16,17]. Thus, YidC is recognized as an insertase; however, most previous studies have been conducted in the absence of DAG and MPIase. Nishiyama et al. reported that substrate proteins were not inserted into DAG-containing liposomes without MPIase, even in the presence of YidC, whereas insertion was dependent on MPIase without YidC. In contrast, when YidC coexists with MPIase in DAG-containing liposomes, it accelerates insertion [18,19]. Therefore, we consider that MPIase functions at the initial step of protein integration into the membrane, whereas YidC completes insertion later.

To date, several MPIase analogs have been synthesized to elucidate insertion mechanisms [20,21]. A minimal structural unit of MPIase, mini-MPIase-3 (Figure 1c), consisting of only one trisaccharide unit, displayed significant activity, although it was less efficient than natural MPIase [21]. Trisac-DAG, a mini-MPIase-3 analog lacking pyrophosphate, and mini-ECA-3, which has a monophosphate linker instead of a pyrophosphate linker (Figure 1c), exhibited lower integration activity than mini-MPIase-3. Structure–activity relationship studies have enabled us to analyze the insertion process at the atomic level using natural MPIase and its synthetic analogs [20,21]. We examined the Sec-independent membrane protein insertion mechanisms facilitated by MPIase based on physicochemical analytical techniques such as solid-state nuclear magnetic resonance (NMR), fluorescence measurements, and surface plasmon resonance (SPR) [22,23,24].

In this review, we describe the mechanism of the initial step of Sec-independent membrane insertion induced by MPIase. Before discussing the role of MPIase, we outline the physicochemical characteristics of membranes that may affect protein membrane insertion. Subsequently, we introduce our results to verify the effects of membrane properties on insertion using physicochemical analytical methods and describe how MPIases support membrane insertion into DAG-containing membranes. Next, we discuss the interactions between MPIase and membrane proteins, which affect the efficiency prior to insertion. Finally, the proposed insertion mechanism facilitated by MPIase is described in detail.

## 2. Factors Influencing Membrane Protein Insertion

Membrane insertion that does not require Sec translocons is affected by the physicochemical characteristics of the membranes. Historically, the membrane insertion of peptides, especially antimicrobial peptides (AMPs), has been extensively studied in various membranes. In this section, we describe the general effects of membrane lipids on alterations in membrane properties.

### 2.1. Charges of Membrane Lipids

Membrane insertion can be affected by the charges of membrane lipids. The polar head of phospholipids commonly has a negatively charged monovalent phosphate diester group. The net charge of the lipids depends on the attached functional group; phosphatidylcholine (PC) and phosphatidylethanolamine (PE), which have positively charged choline and ethanolamine groups, respectively, are neutral, whereas phosphatidylglycerol (PG), which has an uncharged glycerol group, and phosphatidylserine (PS), which has a twitterionic serine residue, are acidic with negative net charges. The lipid composition varies considerably among organisms; eukaryotic cell membranes contain large amounts of PC and cholesterol, whereas bacterial cell membranes are rich in PE and PG. In general, positively charged peptides exhibit a higher insertion ability into negatively charged membranes than into electrically neutral membranes. For example, many positively charged AMPs demonstrate greater binding and insertion into PG-rich membranes than into PC-only membranes [25,26]. This difference is thought to be one of the reasons why antimicrobial peptides selectively disrupt bacterial membranes.

### 2.2. Acyl Chain Ordering

The acyl chain ordering of membrane lipids also influences membrane insertion. In cholesterol-containing membranes, the acyl chains of the membrane lipids are ordered. Since the polar head of cholesterol is a hydroxyl group, which is much smaller than that of phospholipids, cholesterol tends to fit between lipids with large head groups, such as PC. Under the “umbrella” of the phospholipid head groups, the acyl chains of the phospholipid and sterol ring of cholesterol become tightly packed, making insertion difficult [27]. Indeed, the presence of cholesterol has been shown to significantly inhibit peptide membrane binding and insertion [25]. Cholesterol is abundant in mammalian membranes but absent in bacterial membranes. As mentioned later, in *E. coli* membranes, DAG functions in a manner similar to that of cholesterol.

### 2.3. Spontaneous Lipid Curvature

Non-cylindrical lipids in the bilayers tend to modify the physicochemical properties of membranes. When non-cylindrical lipids form a bilayer, their curvature differs from the spontaneous curvature of the lipid. To compensate for this difference, lateral pressure was induced in the membrane. For example, a membrane composed of cone-shaped lipids (lipids with a small head group and larger acyl chain area) increases the lateral pressure in the acyl chain region, whereas that of inverted cone-shaped lipids (lipids with a large head group and smaller acyl chain area) results in higher lateral pressure in the head group region. Alterations in the lateral pressure influence the ability to insert a protein into the membrane. The depth of peptide membrane insertion depends on the spontaneous curvature of the lipids constituting the membrane [28]. In membranes containing lipids with negative spontaneous curvature, peptides bind easily to the membrane surface because of the lower pressure in the region of the head group. However, deeper insertions of peptides become difficult owing to the higher lateral pressure inside the membrane. In contrast, in membranes containing lipids with a positive spontaneous curvature, peptides have difficulty binding to the membrane surface; however, once bound, the peptides can easily penetrate deeper into the bilayer.

In addition, binding and insertion into membranes containing cone-shaped PE depends on the type of peptide. The intervesicular transfer of the hydrophobic transmembrane helix peptide, (AALALAA)_3_, decreased as the PE fraction increased [29]. The tightness of the PE membranes hampered their transfer. For the amphipathic α-helical peptide, Ac-18A-NH_2_, however, there was an increased partitioning of the peptide to the membrane correlating with the rise in the PE mole fraction [30]. In this case, the positively charged side of the amphipathic peptide was in contact with the hydrophilic membrane surface. Although the small head group of PE is advantageous for accepting the peptide, the amphipathic peptide could not be further embedded in the hydrophobic core region and accumulated on the membrane surface.

### 2.4. Membrane Defects

The induction of positive curvature in the membrane causes structural defects. With structural defects, the hydrophobic core of the membrane is more exposed to the membrane surface, making it easier for the hydrophobic side chains of the peptides to interact with the membrane. For example, the amphiphilic helical peptide EpN-18, derived from the N-terminus of epsin-1, induces positive membrane curvature. Coexistence with EpN-18 enhances the plasma membrane permeability of the octa-arginine peptide, possibly because EpN-18 increases structural defects in the membrane, exposing its hydrophobic core to the membrane surface [31,32].

### 2.5. Conformational Changes of Proteins Associated with Membranes

Conformational changes are often observed when peptides bind into membranes. When an amphiphilic peptide is inserted into the membrane, favorable interactions between nonpolar residues and the lipid tail and between charged residues and the solvent molecules or charged sites of the lipid head group convert the peptide from a random coil to a helix. In some cases, amphiphilic peptides are further inserted into the hydrophobic core. Hydrophobic peptides are readily inserted into the hydrophobic core of the membrane and are simultaneously converted to the α-helix structure.

## 3. Effect of Membrane Properties on Membrane Insertion

**Figure 2:**
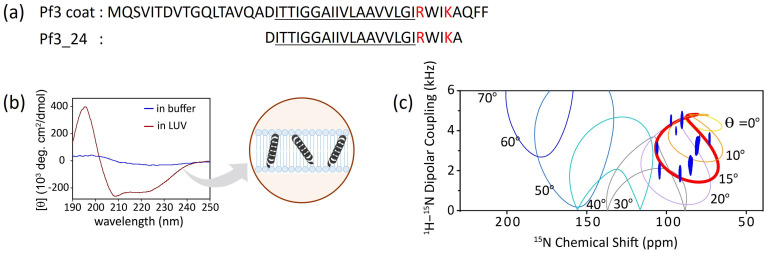
(**a**) Amino acid sequence of Pf3_24 and substructure of Pf3 coat, Pf3_24. Hydrophobic region is underlined, and basic residues are colored in red. (**b**) Circular dichroism (CD) spectra of Pf3_24 in 1,2-dimyristoyl-*sn*-glycero-3-phosphocholine (DMPC) liposomes (dark red) or in buffer (blue) [23]. (**c**) ^1^H–^15^N SAMPI4 spectrum of Pf3_24 in DMPC/1,2-diheptanoyl-*sn*-glycero-3-phosphocholine (DHPC) bicelles [23]. The simulated PISA wheels with varied tilt angles *θ* (0–70°) were overlaid onto the spectrum.

In this section, the verification of the effect of membrane properties on membrane insertion using a Sec-independent substrate is described. Generally, membrane proteins have one or two hydrophobic transmembrane regions and basic amino acid residues at the cytoplasmic loop, which is known as the “positive-inside rule” [33,34]. As a model substrate for Sec-independent integration, we have used the Pf3 coat protein (hereafter referred to as Pf3 coat), a major coat protein of the bacteriophage Pf3, and its derivatives (Figure 2a). To fully penetrate the extramembrane region, the insertion of intact Pf3 coat into the lipid bilayer sometimes requires YidC or a membrane potential. Because we focused on the initial insertion process, truncated Pf3 coat (Pf3_24) corresponding to the transmembrane region was used as the simplest model for verification.

### 3.1. Conformational Changes and Topology of Pf3_24 in Membranes

Typically, the transmembrane regions of membrane proteins are inclined to adopt α-helical or β-strand conformations. Circular dichroism (CD) spectra showed that Pf3_24 adopts an α-helical conformation when inserted into the lipid bilayer (Figure 2b, dark red), although it is difficult to determine the secondary structure of Pf3_24 in buffer solution due to its tendency to form aggregates and precipitates (Figure 2b, blue). Solid-state NMR is a useful technique for obtaining the accurate tilt angles of helical peptides or helical parts of proteins with respect to the membrane. The topology of Pf3_24 in bicelles was determined using ^1^H–^15^N SAMPI4 spectra (Figure 2c) [35]. A characteristic PISA wheel pattern of Pf3_24 (Figure 2c, blue) implies the formation of an α-helical structure in bicelles [36]. The insertion angle of Pf3_24 into the membrane from the bilayer normal was determined to be approximately 15° by fitting to account for the wobbling motion of the bicelles. Based on its angle and hydrophobicity, Pf3_24 directly accessed the deeper regions of the membrane. Simultaneously, conversion to the α-helix structure likely occurs.

### 3.2. Insertion of Pf3_24 into Membranes

**Figure 3:**
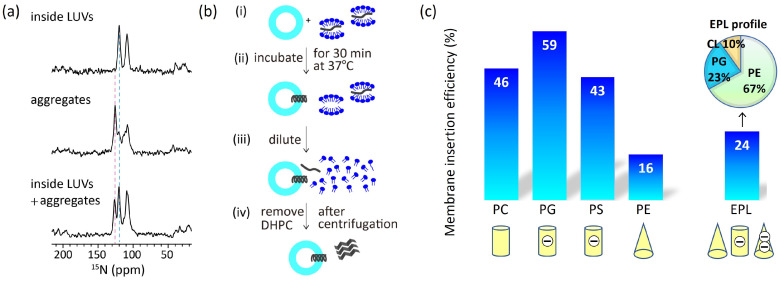
(**a**) ^15^N cross polarization under magic angle spinning (CPMAS) NMR spectra of Pf3_24 [23]. (top) Pf3_24 was fully reconstituted into DMPC LUVs. (middle) Pf3_24 in aggregates. The sample was prepared without the use of LUVs in the procedure shown in (**b**). (bottom) The sample was prepared using the procedure shown in (**b**) for DMPC LUVs. The pink dashed line shows the chemical shift for the β-strand conformation, and the light blue dashed line shows that for α-helix and/or random-coil conformations. The signal observed at approximately 110 ppm originated from glycine. (**b**) Procedure for inserting Pf3_24 into the membranes. (i) Pf3_24 (dark gray) solubilized in DHPC solution was mixed with LUVs composed of various lipid types. (ii) The mixture was incubated for 30 min at 37 °C. (iii) Then, it was diluted with a buffer solution until the DHPC concentration reached below its critical micelle concentration. (iv) The supernatant containing DHPC was removed by centrifugation. Precipitates containing both LUVs and aggregates were subjected to ^15^N CPMAS NMR. (**c**) Membrane insertion efficiency of Pf3_24, determined using the procedure shown in (**b**), into several types of membranes.

We estimated insertion efficiency based on the strong correlation between ^15^N NMR chemical shifts and protein secondary structure [37,38]. It was reported that valine residues take the averaged ^15^N chemical shift values 123.27 ppm, 119.66 ppm, and 119.53 ppm for the β-strand, random coil, and α-helix, respectively [37]. As aforementioned, Pf3_24 has a complete α-helical structure in large unilamellar vesicles (LUVs) of 1,2-dimyristoyl-*sn*-glycero-3-phosphocholine (DMPC) (Figure 3a, top), which was verified by the chemical shift value for valine. Conversely, in the absence of membranes, Pf3_24 formed aggregates, yielding a stronger signal at 125 than at 120 ppm, which indicate that most of it adopted β-strand-rich structures, whereas some parts formed random coils and α-helical structures (Figure 3a, middle). To observe membrane insertion, as shown in Figure 3b, Pf3_24 solubilized in 1,2-diheptanoyl-*sn*-glycero-3-phosphocholine (DHPC) solution was mixed with LUVs composed of various lipid types. During incubation, some Pf3_24 was inserted into the LUVs, whereas the remainder formed aggregates outside the LUVs. The third spectrum was obtained from the experiment conducted in the presence of DMPC LUVs (Figure 3a, bottom). We considered that the third spectrum represents the weighted sum of the first and second spectra, with the coefficient of the first spectrum corresponding to the insertion efficiency. Thus, the relative peak intensities corresponding to the α-helix and the aggregates in the third spectrum provided an insertion efficiency of 46% for the DMPC membrane. Similarly, we calculated the insertion efficiency values for membranes with various lipid compositions (Figure 3c). However, this approach is applicable for each membrane system when the protein forms a complete α-helical conformation in the membrane and a β-sheet rich conformation outside the membrane with a constant ratio of conformations in the aggregates.

In comparing the efficiencies of the major membrane lipids, we noted significantly lower levels in PE and EPL membranes, which contained cone-shaped lipids. Thus, the membrane insertion efficiency depends more on the molecular shape than on the charge of the lipids.

### 3.3. Acyl Chain Mobility and Membrane Surface Packing

**Figure 4:**
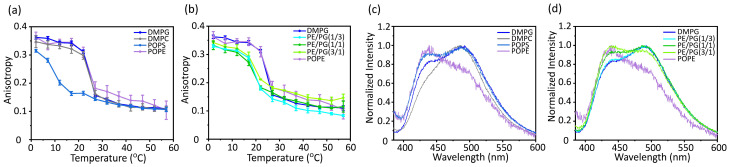
Fluorescence anisotropy of DPH (**a**,**b**) and fluorescence emission spectra of Laurdan at 37 °C (**c**,**d**) incorporated into various types of membranes [23]. DMPG, 1,2-dimyristoyl-*sn*-glycero-3-phospho-(1′-rac-glycerol); DMPC, 1,2-dimyristoyl-*sn*-glycero-3-phosphocholine; POPS, 1-palmitoyl-2-oleoyl-*sn*-glycero-3-phospho-L-serine; POPE, 1-palmitoyl-2-oleoyl-*sn*-glycero-3-phosphoethanolamine.

The mobility inside the membrane was analyzed by measuring the anisotropy of the rod-shaped fluorescent probe 1,6-diphenyl-1,3,5-hexatriene (DPH), which was incorporated near the bilayer core region (Figure 4a,b). DPH exhibits strong polarization characteristics in membranes due to its shape, and these polarization characteristics are influenced by the membrane environment. An increase in the fluorescence anisotropy values of DPH reflects an increase in the molecular order in the membrane core region [39]. The membrane mobility of PE was significantly lower than that of the other bulk lipids (Figure 4a). In addition, the membrane mobility decreased as the PE concentration increased (Figure 4b).

Membrane packing was also analyzed by measuring the emission spectra of Laurdan, which resides around the hydrophilic–hydrophobic interface of the lipid bilayers (Figure 4c,d) [40]. The emission spectrum of Laurdan is sensitive to the water content of the surrounding environment. The red shift of Laurdan’s emission spectrum indicates more hydration by loosening the lipid packing, whereas the blue shift of the spectrum indicates membrane tightening. The generalized polarization (GP) values [*GP* = (*I*_440_ − *I*_490_)/(*I*_440_ + *I*_490_)] express the degree of hydration. The membranes composed of the cone-shaped lipid PE showed the strongest tightening of membrane packing (Figure 4c). Furthermore, the membrane packing became remarkably tight as the PE concentration increased (Figure 4d).

### 3.4. Correlation between Membrane Physicochemical Properties and Membrane Insertion Efficiency of Pf3_24

**Figure 5:**
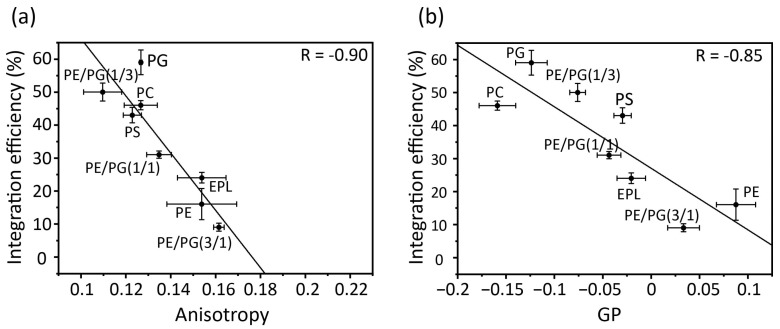
Correlation between membrane insertion efficiency values and fluorescence anisotropy values of DPH at 37 °C (**a**) and Laurdan GP values, *GP* = (*I*_440_ − *I*_490_)/(*I*_440_ + *I*_490_), (**b**) in various types of membranes. Global linear fits were performed for all bulk phospholipids.

Correlation plots of acyl chain mobility (DPH anisotropy) and membrane surface packing (Laudan GP) in various membranes against insertion efficiency (Figure 5a,b) indicate the contribution of physicochemical properties to membrane insertion. Both the anisotropy and GP values strongly correlated with the membrane insertion efficiency. Thus, in membranes composed only of bulk phospholipids, acyl chain mobility and membrane packing play direct roles in insertion, with regression lines serving as reliable guidelines. For example, proteins are readily inserted into loosely packed membranes formed by cylindrical lipids, such as PC, PG, and PS. However, as the concentration of PE increased, the acyl chain mobility decreased and the surface packing tightened, leading to the suppression of insertion. PE easily results in negative curvature stress in membranes owing to its small head group, which makes it difficult for the protein to interact with the core of the membranes.

## 4. Role of MPIase on Membrane Protein Insertion

Thus far, we have evaluated the insertion of Pf3_24 into membranes composed only of bulk lipids. However, in actual *E. coli* inner membranes, the presence of minor endogenous components, DAG and MPIase, efficiently regulates membrane protein insertion. Spontaneous membrane insertion is blocked by DAG, whereas suppressed insertion is recovered by MPIase. In our study, at physiological concentrations, neither MPIase nor DAG destabilized the lipid bilayer structure or caused membrane fusion [24]. Thus, the membrane insertion of proteins supported by MPIase is not accompanied by membrane disruption, as is the case with antimicrobial peptides. DAG is a small cone-shaped lipid, whereas MPIase is an inverted cone-shaped glycolipid containing a DAG substructure and an additional head group consisting of a long sugar chain and pyrophosphate. Owing to their unique molecular structures compared to other membrane lipids, they exert distinct effects on membrane properties. In this section, we describe the mechanism by which MPIase supports membrane protein insertion into *E. coli* membranes in the presence of DAG [23].

### 4.1. Mobility of MPIase in Membranes

**Figure 6:**
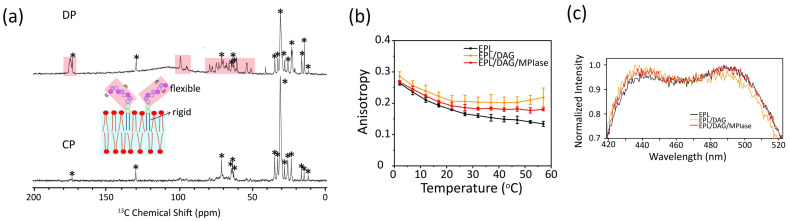
(**a**) Direct polarization (DP) (top) and cross-polarization (CP) (bottom) ^13^C NMR spectra of EPL liposomes in the presence of uniformly ^13^C-labeled natural MPIase (5 wt%) under MAS with a spinning speed of 5 kHz [24]. The sugar chain signals that appeared only in the DP spectrum are shown in salmon pink. The peaks from EPL are marked with *. (**b**,**c**) Fluorescence anisotropy of DPH (**b**) and fluorescence emission spectra of Laurdan at 37 °C (**c**) incorporated into EPL-based membranes [23].

The relative molecular mobility of MPIase and bulk membrane lipids was examined by measuring the ^13^C cross-polarization (CP) and direct polarization (DP) NMR spectra of EPL liposomes containing uniformly ^13^C-labeled natural MPIase (5 wt%) using magic angle spinning (MAS) (Figure 6a). It is generally known that NMR signals of all components are observed using DP, whereas those of rigid ones are selected by the dipolar transfer in CP. A comparison of the relative signal intensity observed in the DP (Figure 6a, top) and CP (Figure 6a, bottom) spectra suggests a higher mobility of the MPIase sugar chain than its lipid part and the other lipids in the liposomes. Higher fluctuation of sugar chain head groups compared to its lipid moiety has also been observed in other glycolipids [41,42,43], and generally, longer sugar chains amplify this effect [44]. Moreover, in the ^31^P T1 values of the head group of bulk lipids, fluctuation of the MPIase sugar chain increased their movement. DAG had little influence on the lipid head group mobility because its small head groups were not exposed to the membrane surface [24].

### 4.2. Effects on Flip–Flop Motion of DAG

DAG has a very rapid flip–flop rate compared to phospholipids owing to their compact shape [45]. The rapid flip–flop motion of DAG likely disturbs membrane protein insertion. Once a transient defect is formed among phospholipids in the membrane, DAG can flip across the lipid bilayer, filling the space and reducing unfavorable exposure to the hydrophobic acyl chains. This weakens the contact of proteins with the inside of the membrane and blocks the insertion of membrane proteins. In contrast, the flip–flop is diminished in the presence of mini-MPIase-3 [24]. Locally, unfilled hydrophobic spaces are formed more frequently and are retained longer, allowing proteins to insert more easily into the membrane.

### 4.3. Effects of DAG and MPIase on Acyl Chain Mobility and Membrane Surface Packing

Acyl chain mobility in the EPL membrane core region was analyzed using the anisotropy of DPH (Figure 6b). The acyl chain mobility was significantly reduced by DAG. However, MPIase partially restored the reduced mobility of the DAG-containing membranes. Lipid acyl chain ordering was also studied by measuring the ^2^H NMR spectra. Quadrupole splitting increased in the presence of DAG but returned to the original levels after the addition of natural MPIase (5 wt%) into the DAG-containing liposomes. Thus, DAG promotes the ordering of lipid acyl chains, whereas MPIase compensates for the membrane ordering induced by DAG. The increased packing of the hydrophobic segment by DAG further reduced lateral lipid diffusion. Similar to cholesterol, DAG fits under the polar head groups of lipids, enhancing the order of acyl chains. However, MPIase rebalances the head group and acyl chain volumes of lipids in the membranes and releases the packing pressure inside the membrane.

Membrane surface packing was analyzed by measuring the emission spectra of Laurdan (Figure 6c). DAG slightly tightened the membrane packing, whereas MPIase partially loosened the packing in DAG-containing membranes. Thus, MPIase counteracted the effects of DAG on membrane properties, both in the membrane core region and on the surface. However, the influence of DAG and MPIase on the membrane surface region was less than that on the membrane core region, suggesting that access to the membrane core region is more critical than initial contact with the surface region for successful protein insertion.

**Figure 7:**
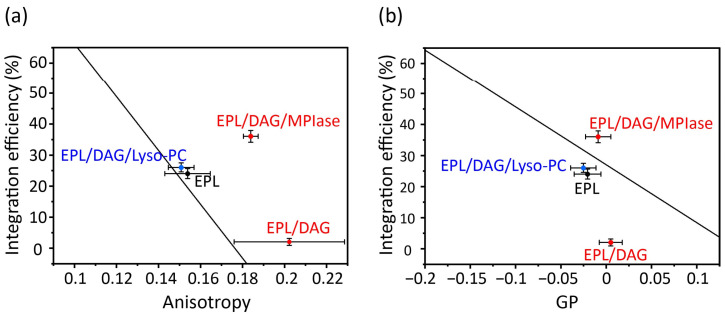
Correlation between membrane insertion efficiency values and fluorescence anisotropy values of DPH at 37 °C (Figure 6b) (**a**) and membrane insertion efficiency values and Laurdan GP values at 37 °C calculated from the emission spectra (Figure 6c) (**b**) in the EPL-based membranes. The black line shows the regression line obtained from the global linear fits performed on the bulk phospholipids in Figure 5.

The correlation plots clearly demonstrate how alterations in the physicochemical properties of the lipid membranes containing DAG and MPIase contribute the insertion of proteins into EPL membranes (Figure 7). In the presence of DAG (5 mol% to EPLs), the mobility of the core region was dramatically reduced (Figure 7a); however, the mobility of the membrane surface region was not significantly reduced (Figure 7b). Therefore, we conclude that the drastic inhibition of insertion by DAG is attributable to decreased mobility of the core region. Moreover, we considered that the high insertion efficiency of MPIase was due, at least partly, to compensation for the effects in shape. Thus, we compared MPIase with lyso-PC, which has an inverse-cone shape similar to that of MPIase, because it is known that the presence of lyso-PC enhances the insertion of antimicrobial peptides in PC membranes [46]. The addition of lyso-PC (5 mol%) to the DAG-containing EPL membrane restored both the membrane mobility and the insertion efficiency of Pf3_24. The correlation is on the regression line, indicating that the effect of lyso-PC on insertion can be explained only by the alteration of membrane physicochemical properties. Conversely, after adding MPIase (1 mol%) to the DAG-containing EPL membrane, the insertion efficiency was much higher than what was expected from the correlation plot against anisotropy values. Therefore, factors other than membrane properties should also support the high insertion efficiency in the presence of MPIase. To identify other causes, we focused on the intermolecular interactions between MPIase and substrate proteins.

### 4.4. Identification of the Contact Sites by Using Docking Simulations

**Figure 8:**
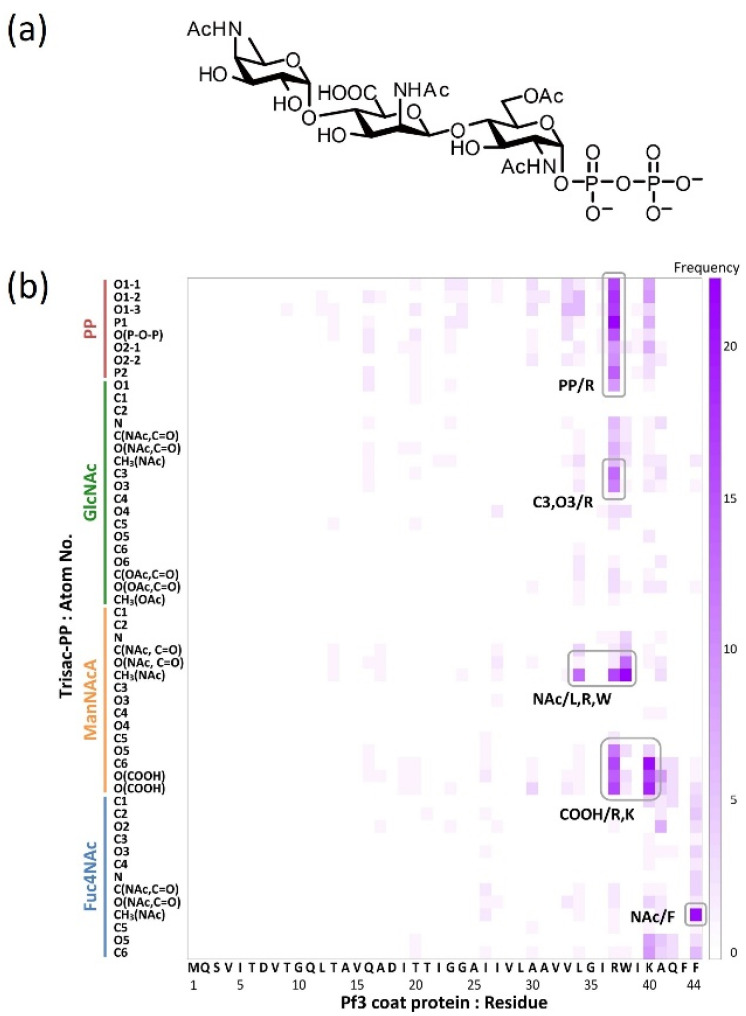
(**a**) Molecular structure of Trisac-PP. (**b**) Heat map of frequencies in the docking simulation. It illustrates the frequencies of interaction between each atom of Trisac-PP and each amino acid residue of Pf3 coat [22].

The functional groups required for molecular interactions between MPIase and the protein were estimated by docking simulations using the MOE software, version 2019.0102 [47]. Docking simulations of pyrophosphorylated trisaccharide moieties, Trisac-PP, with Pf3 coat suggested the hydrophobic interactions between the acetyl groups in MPIase and the hydrophobic residues of Pf3 coat and electrostatic interactions between the pyrophosphate or carboxylate of MPIase and the basic amino acid residues of Pf3 coat (Figure 8). The reduction in contact in all combinations observed when using MPIase analogs lacking 6-*O*-acetyl of GlcNAc or pyrophosphate emphasizes the importance of these functional groups.

### 4.5. Binding Kinetics and Affinity of MPIase−Pf3 Coat Interactions

**Figure 9:**
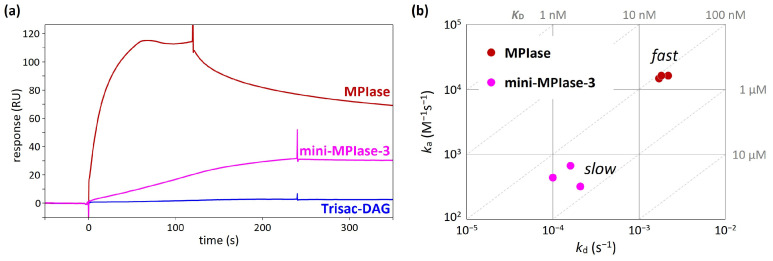
(**a**) SPR sensorgrams of the binding of natural MPIase, mini-MPIase-3, and Trisac-DAG to Pf3 coat [22]. (**b**) The on–off rate map showing the kinetic profiles of the interaction of MPIase and mini-MPIase-3 with Pf3 coat [22].

In an integration activity assay using in vitro translation [48], MPIase with a long sugar chain showed much higher activity than mini-MPIase-3 [21]. In addition, Trisac-DAG, a mini-MPIase-3 analog without a pyrophosphate group, exhibited much lower insertion than mini-MPIase-3 [20]. These differences in insertion activity can be understood by analyzing binding kinetics and affinity using SPR [49,50]. In the SPR measurements, the glycolipid analytes flowed over the flow cells on which Pf3 coat had been immobilized. While direct comparisons of response levels are unsuitable because of notable variations in molecular weight among ligands, the comparison of responses induced by MPIase, mini-MPIase-3, and Trisac-DAG highlights the involvement of pyrophosphate in the interaction of MPIase-Pf3 coat (Figure 9a) [22]. The on–off rate map, in which the association (*k*_a_) and dissociation (*k*_d_) rates determined by fitting analyses were plotted, clearly demonstrated a greater contribution of the long sugar chain of MPIase to rapid contact with Pf3 coat (Figure 9b). This is likely the reason for the higher integration activity of MPIase compared with that of mini-MPIase-3.

### 4.6. Interaction of the MPIase Pyrophosphate Group with Basic Residues in Pf3_27

**Figure 10:**
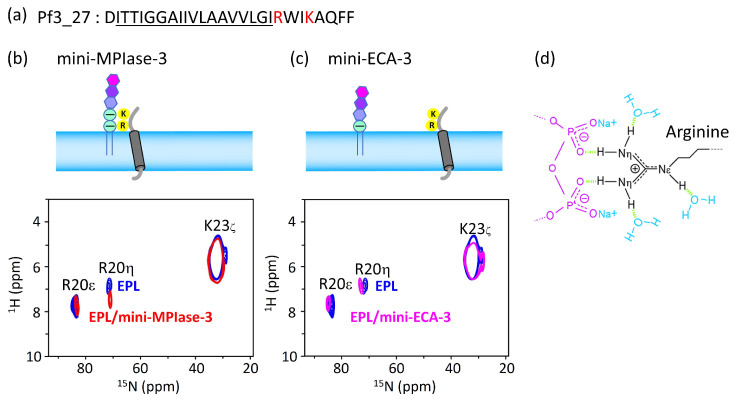
(**a**) Amino acid sequence of substructure of Pf3 coat, Pf3_27. Hydrophobic region is underlined, and basic residues are colored in red. (**b**,**c**) Arg and Lys side-chain signal regions of the 2D ^1^H-^15^N FSLG-HETCOR spectrum of Pf3_27 in EPL membranes in the presence of mini-MPIase-3 ((**a**), blue) [23] or mini-ECA-3 ((**b**), pink) and absence of them ((**a**,**b**), blue). (**d**) Schematic models of Arg side-chain (black) interaction with the pyrophosphate linker part of MPIase (purple). Light green dots show hydrogen bonding.

As can be seen from the results of the docking simulations and SPR experiments, the MPIase pyrophosphate linker is one of the key structures involved in membrane insertion activity. The ^1^H-^15^N FSLG-HETCOR spectrum of Pf3_27, which has a transmembrane region followed by cytoplasmic residues (Figure 10a), exhibited a selective interaction between the pyrophosphate in MPIase and Arg residues (Figure 10b–d). The signal of the R20 H_η_ protons was shifted 0.53 ppm downfield in the presence of mini-MPIase-3 (Figure 10b), whereas the proton signal did not change with mini-ECA-3 that has a monophosphate linker (Figure 10c) [20,23]. Generally, basic residues in membrane proteins are abundant near the membrane surface on the cytoplasmic side; this is known as the positive-inside rule [33,34]. Therefore, the double negatively charged pyrophosphate group of MPIase attracts basic residues to the membrane surface (Figure 10d). The downfield shift value of 0.53 ppm due to hydrogen bonding of Pf3_27 with MPIase is smaller than that observed for the ligand–receptor complex (about 2–3 ppm) [23,51]. Therefore, the binding of the guanidino group of R20 to the pyrophosphate of MPIase is weaker than that in the ligand–receptor complex and more transient. Transient binding allows MPIase to detach the protein after insertion into the membrane.

### 4.7. Inhibition of Protein Aggregation by MPIase

**Figure 11:**
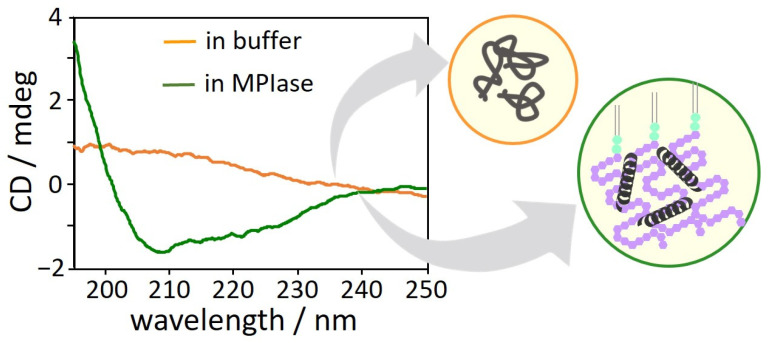
CD spectra of Pf3 coat in buffer in the presence (green) and absence (orange) of MPIase [22].

The interactions between MPIase and Pf3 coat induce changes in the secondary structure of Pf3 coat [22]. The CD spectra of Pf3 coat clearly exhibited that without MPIase, Pf3 coat did not form a definite secondary structure (Figure 11, orange), while it exhibited mostly an α-helical structure with MPIase (Figure 11, green). The long sugar chain of MPIase is likely to capture nascent proteins through multiple interactions, such as the acetyl groups of MPIase and the hydrophobic region of the proteins or the carboxyl groups of MPIase and the basic amino acid residues of the proteins, which prevent the protein from aggregating. Therefore, MPIase might act as a chaperone during membrane insertion. In this case, to effectively inhibit protein aggregation, MPIase might form a self-aggregated structure on the inner membrane of *E. coli*.

## 5. Mechanism of Sec-Independent Membrane Protein Insertion by MPIase

**Figure 12:**
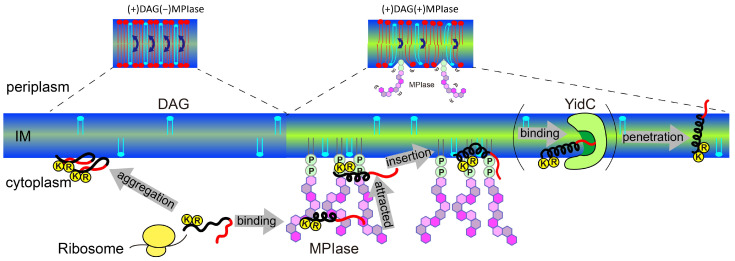
Schematic model of the membrane insertion of a small hydrophobic substrate protein (black: hydrophobic region, red: hydrophilic region) supported by MPIase in *E. coli* membranes.

Finally, the insertion mechanism of small hydrophobic proteins which contain basic amino acids at their C-terminus, like Sec-independent membrane protein, into *E. coli* membranes under the regulation of MPIase and DAG is summarized in Figure 12.

1. DAG blocks spontaneous insertion by reducing acyl chain mobility in the membrane core. The rapid flip–flop of DAG also reduces lateral diffusion and fills membrane defects, exposing acyl chains and making it difficult for proteins to contact the inside of the membranes. However, the presence of MPIase cancels out the effect of DAG and restores mobility. The flexible bulky sugar chain of MPIase makes the membrane surface flexible, increases the mobility of the membrane core region, and reduces the flip–flop motion of DAG. Small hydrophobic membrane proteins preferentially associate with the membrane core region rather than with the aqueous environment outside the membrane.
2. After a protein is released from a ribosome, the long sugar chain of MPIase captures the protein and prevents its aggregation. The protein efficiently changes its conformation through rapid association with and dissociation from the flexible long sugar chains of MPIase.
3. The basic residues that generally exist in the cytoplasmic loops near the hydrophobic transmembrane regions of the protein are attracted towards the membrane surface through electrostatic interactions with the pyrophosphate of MPIase.
4. The protein easily gains access to the loosened membrane core through hydrophobic interactions and finally integrates into the membrane.

MPIase interacts with proteins via its pyrophosphate group. However, this binding is weak, and MPIase can easily detach from the protein. It is likely that the protein is then delivered to YidC in the inner membrane. The detached MPIase can assist another substrate that has not yet been inserted into the membrane. Thus, MPIase efficiently supports membrane insertion.

## 6. Conclusions

This study discusses the various membrane properties that affect Sec-independent membrane insertion and the contributions of MPIase and DAG to membrane properties. Both MPIase and DAG are minor components of *E. coli* membranes. However, by acting in opposite directions, they effectively alter membrane properties. Comprehensive analysis of the membrane insertion activity, physicochemical properties, and molecular interactions between MPIase and its substrates provides an understanding of the atomic-level molecular mechanisms by which MPIase prevents substrate protein aggregation, attracts substrates near the membrane surface, and promotes membrane insertion. These findings reveal novel biological roles of glycolipids. Since MPIase is also involved in other protein translocation pathways across membranes [7,11], further studies on MPIase clustering on the membrane and its distribution in cells will provide insights into the diverse functions of MPIase, including membrane transport.

## Data Availability

No new data are reported in this review article.

## Abbreviations

The following abbreviations are used in this manuscript:

membrane protein integrase	MPIase
*Escherichia coli*	*E. coli*
diacylglycerol	DAG
*E. coli* phospholipids	EPLs
phosphatidylethanolamine	PE
phosphatidylglycerol	PG
cardiolipin	CL
antimicrobial peptides	AMPs
phosphatidylcholine	PC
phosphatidylserine	PS
1,2-dimyristoyl-*sn*-glycero-3-phosphocholine	DMPC
1,2-diheptanoyl-*sn*-glycero-3-phosphocholine	DHPC
large unilamellar vesicles	LUV
cross-polarization under magic angle spinning	CPMAS
1,6-diphenyl-1,3,5-hexatriene	DPH
generalized polarization	GP
1,2-dimyristoyl-*sn*-glycero-3-phospho-(1′-rac-glycerol)	DMPG
1-palmitoyl-2-oleoyl-*sn*-glycero-3-phospho-L-serine	POPS
1-palmitoyl-2-oleoyl-*sn*-glycero-3-phosphoethanolamine	POPE
magic angle spinning	MAS
surface plasmon resonance	SPR
association rate	*k*_a_
dissociation rate	*k*_d_
circular dichroism	CD
